# The retinoid X receptor α modulator K-80003 suppresses inflammatory and catabolic responses in a rat model of osteoarthritis

**DOI:** 10.1038/s41598-021-96517-y

**Published:** 2021-08-20

**Authors:** Hua Li, Xiaofan Li, Boyu Yang, Junnan Su, Shaofang Cai, Jinmei Huang, Tianfu Hu, Lijuan Chen, Yaping Xu, Yuhang Li

**Affiliations:** 1 The Department of Science and Education, The Second Affiliated Hospital of Xiamen Medical College, Xiamen, China; 2 Hematopoietic Stem Cell Transplantation Center, Fujian Institute of Hematology, Fujian Provincial Key Laboratory On Hematology, Department of Hematology, Fujian Medical University Union Hospital, No. 29 Xinquan Street, Gulou District, Fuzhou, 350001 China; 3 The Department of Orthopedics, The Second Affiliated Hospital of Xiamen Medical College, Xiamen, China; 4 The Department of Hematology and Rheumatology, The Second Affiliated Hospital of Xiamen Medical College, Xiamen, China; 5 Department of Traditional Chinese Medicine, Community Health Service Center of Qiaoying Street, Xiamen, China; 6 Key Laboratory of Functional and Clinical Translational Medicine, Fujian Province University, Xiamen Medical College, Xiamen, China; 7 CAS Key Laboratory of Design and Assembly of Functional Nanostructures, and Fujian Provincial Key Laboratory of Nanomaterials, Fujian Institute of Research On the Structure of Matter, Chinese Academy of Sciences, Fuzhou, China; 8 Xiamen Institute of Rare-Earth Materials, Haixi Institutes, Chinese Academy of Sciences, Fujian, 361005 China

**Keywords:** Pharmacology, Osteoimmunology

## Abstract

Osteoarthritis (OA), a most common and highly prevalent joint disease, is closely associated with dysregulated expression and modification of RXRα. However, the role of RXRα in the pathophysiology of OA remains unknown. The present study aimed to investigate whether RXRα modulator, such as K-80003 can treat OA. Experimental OA was induced by intra-articular injection of monosodium iodoacetate (MIA) in the knee joint of rats. Articular cartilage degeneration was assessed using Safranin-O and fast green staining. Synovial inflammation was measured using hematoxylin and eosin (H&E) staining and enzyme-linked immunosorbent assay (ELISA). Expressions of MMP-13, ADAMTS-4 and ERα in joints were analyzed by immunofluorescence staining. Western blot, RT-PCR and co-Immunoprecipitation (co-IP) were used to assess the effects of K-80003 on RXRα-ERα interaction. Retinoid X receptor α (RXRα) modulator K‐80003 prevented the degeneration of articular cartilage, reduced synovial inflammation, and alleviated osteoarthritic pain in rats. Furthermore, K-80003 markedly inhibited IL-1β‐induced p65 nuclear translocation and IκBα degradation, and down-regulate the expression of HIF-2α, proteinases (MMP9, MMP13, ADAMTS-4) and pro-inflammatory factors (IL-6 and TNFα) in primary chondrocytes. Additionally, knockdown of ERα with siRNA blocked these effects of K-80003 in chondrocytes. In conclusion, RXRα modulators K-80003 suppresses inflammatory and catabolic responses in OA, suggesting that targeting RXRα‐ERα interaction by RXRα modulators might be a novel therapeutic approach for OA treatment.

## Introduction

Osteoarthritis (OA), the most common degenerative joint disease, is a leading cause of physical disability in the aging population. OA is associated with cartilage damage, inflammation of the synovial membrane, and chronic pain. Articular cartilage destruction is the primary concern in OA^1^. During the progression of OA, the expressions of matrix metalloproteinase 13 (MMP-13) and aggrecanase-1 (ADAMTS-4) in articular chondrocytes are increased, leading to loss of collagens and proteoglycans, and cartilage damage^1^. Moreover, several pro-inflammatory cytokines, including interleukin-1β (IL-1β), interleukin-6 (IL-6), and tumor necrosis factor-α (TNF-α), are also involved in the degeneration of articular cartilage in joint^2^. The etiology and pathogenesis underlying OA are still largely unknown. Nuclear factor-κB (NF-κB) pathways have been shown to be among the major contributors to OA pathology^3^. Targeting these signaling pathway are beneficial in suppressing inflammatory and destructive responses in OA.

Till date, there is no ideal pharmacological interventions for OA, especially for preventing the loss of cartilage^2^. Although nonsteroidal anti-inflammatory drugs (NSAIDs) or a combination of steroid and hyaluronic acid (HA) can reduce joint pain and inflammation, a series of unwanted side effects restrict their application^4^. Anti-cytokine drugs also have been proposed as promising therapeutic agents for OA. These drugs can prevent the progression of joint structural changes, and some of them even have undergone clinical trials^2^. However, the effects of anti-cytokine drugs in OA patients are paradoxical and controversial^2^. Some clinical studies using anti-cytokine drugs were stopped because of toxicity. While platelet-rich plasma or mesenchymal stem cell have been used to treat OA, the high cost and complicated preparation process limited their dissemination to a large amount of OA patients^5,6^. It is still highly desirable to develop new therapeutic approaches that can effectively maintain cartilage homeostasis while attenuate inflammation and alleviate pain.

The nuclear receptor (NR) superfamily plays important roles in various cellular processes^7^. Articular cartilage expresses many NRs, including estrogen receptor α (ERα) and retinoid X receptor α (RXRα)^8^. ERα has been proposed as a potential therapeutic target in OA, and drugs (bazedoxifene and raloxifene) regulating Erα functions are potential therapeutic agents for OA^9,10^. RXRα regulates inflammatory responses in different cell types^11^. Dysregulated expression and modification of RXRα is closely associated with various inflammation-related diseases, including OA^8,12^. However, the role of RXRα in OA conditions and whether RXRα modulator can treat OA is still largely unknown. K‐80003, a sulindac analog with enhanced RXRα binding affinity (IC_50_ = 2.4 μM) and diminished COX inhibitory potency (IC_50_ > 1 mM), exhibits profound anti-inflammatory effects in macrophages in colorectal carcinogenesis^12,13^. We speculated that K‐80003 may have beneficial effects for OA. Thus, in the present study, we investigated whether RXRα modulator K‐80003 could attenuate the development of OA. Our results suggested that K‐80003 prevented the degeneration of articular cartilage by interrupting RXRα-ERα interaction, subsequently enhancing ERα signaling and suppressing the NF‐κB pathway.

## Materials and methods

### Materials

All reagents used in the present study were purchased from Sigma-Aldrich (Shanghai, China), seeking the highest grade commercially available unless otherwise indicated.

### Animal experiments

All animal experiments were performed in accordance with Guide and Care and Use of Laboratory Animals from National Institutes of Health (NIH) and ARRIVE, and approved by the Animal Care and Use Committees of Xiamen Medical College in China (Approval No. FJMU IACUC 2020-0109). Total 32 male Sprague-Dawley (SD) rats (200–230 g) were purchased from laboratory animal center of Xiamen university, and maintained at 20–25 °C in a 12 h light/dark cycle. All animals were sacrificed 28 days after MIA injection, and knee joints were dissected for histopathological studies unless otherwise indicated. The time window for the K-80003 (Sigma, 557451) administration was determined in the pharmacokinetic studies. All experiments were designed to generate groups of equal size (n = 8). Experimental data were analyzed using randomization and blind data analysis, and no data were excluded in any experiment. Animals were group-housed in ventilated cages with free access to food and water and allowed to acclimate to the facility. Animal were divided into the following experimental groups:

#### Sham group

Total n = 8 rats were randomly grouped, anesthetized with chloral hydrate (300 mg/kg, i.p.) and injected intra-articularly (i.a.) with saline (20 μL) in the hind knees, followed by i.a. treatment with vehicle (saline with 5% polyethylene glycol 400 and 5% tween 80) at days 7, 10, 14, 17, 21, 24 after MIA treatment.

#### *OA*+ *vehicle group*

Total n = 8 rats were randomly grouped, anesthetized with chloral hydrate (300 mg/kg, i.p.) and injected i.a. with monosodium iodoacetate (MIA) (1 mg in 20 μL saline) in the hind knees, followed by i.a. treatment with vehicle at days 7, 10, 14, 17, 21, 24 after MIA treatment.

#### *OA*+ *K-80003 (1 mg/kg) group*

Total n = 8 rats were randomly grouped, anesthetized with chloral hydrate (300 mg/kg, i.p.) and injected i.a. with MIA (1 mg in 20 μL saline) in the hind knees, followed by i.a. treatment with K-80003 (1 mg/kg initial animal weights, 5% polyethylene glycol 400 and 5% tween 80) at days 7, 10, 14, 17, 21, 24 after MIA treatment.

#### *OA*+ *K-80003 (2 mg/kg) group*

Total n = 8 rats were randomly grouped, anesthetized with chloral hydrate (300 mg/kg, i.p.) and injected i.a. with MIA (1 mg in 20 μL saline) in the hind knees, followed by i.a. treatment with K-80003 (2 mg/kg initial animal weights, 5% polyethylene glycol 400 and 5% tween 80) at days 7, 10, 14, 17, 21, 24 after MIA treatment.

### Histology

Rat chondrocytes were fixed with 4% formaldehyde followed by blocking with goat serum in 0.3M glycine in PBS at room temperature for 1 h. Rat knee joints were fixed for 48 h in 2% formaldehyde, decalcified for 40 days in EDTA (10%, pH 7.5), paraffin embedded and cut into 5-μm-thick sections^14^. Sagittal-oriented sections of the joint medial compartment were processed for immunofluorescence and safranin O and fast green staining. We calculated Mankin scores of cartilage degeneration and synovitis score of the synovium as previously described^15,16^. All of the histology samples were scored blindly and independently by at least two investigators.

### Immunofluorescence

Immunofluorescent staining was performed using a standard protocol^15^. Sections were incubated overnight at 4 °C in the presence of primary antibodies (MMP13, Abcam, ab219620, dilution 1:400; ADAMTS-4, Abcam, ab185722, dilution 1:300; ERα, Santa Cruz, sc-8005, dilution 1:200; p65, Abcam, ab16502, dilution 1:800). Sections were washed with 0.1 M PBS, followed by incubating with secondary antibodies conjugated with Alexa Fluor 488 (Abcam, ab150077, dilution 1:200) or 555 (Abcam, ab150078, dilution 1:200) at room temperature for 1 h while avoiding light. After an additional rinse, the sections were counterstained with DAPI (Vector Lab, dilution 1:1000, Shanghai, China) and observed under confocal microscopy (Olympus, Japan). To confirm the antibody binding specificity for p65, MMP13, ADAMTS-4 and ERα, some sections were also incubated with primary or secondary antibody only. The numbers of MMP13^+^, ADAMTS4^+^ and ERα^+^ cells were automatically counted using Image J software. Total three sections per animal were analyzed. Cells number was calculated from three randomly chosen and non-overlapping fields (460 × 460 µm) in the articular cartilage of each section.

### Enzyme-Linked Immunosorbent Assay (ELISA)

Synovial membranes were collected and homogenized in ice-cold saline solution. The protein levels of IL-1β, IL-6 and TNF-α were examined by the appropriate ELISA kits (Abcam, ab100785, ab100772 and R&D, RLB00) followed the manufacturer’s instructions.

### Behavioral tests

Behavioral tests were performed in a quiet room. Tactile allodynia were measured five times at each time point for the OA knee joints as previously described^17^. Tactile allodynia was tested on the hind paw of rats at day 8, 15, 22 and 28, using a dynamic plantar aesthesiometer (Ugo Basile, Comerio, Italy). Rats were placed on a metal mesh surface in a chamber and allowed to acclimatize to the facility for 15 min prior to testing. The plastic monofilament touched the paw, gradually increasing the force on the plantar until the rat removed its paw, and measured the latency to withdraw the hind paw from the monofilament. The paw withdrawal mechanical thresholds in grams were measured automatically.

### Cell culture and treatment

Rat primary chondrocytes were obtained and cultured using methods as previously described by our group^15,18^. Passage 3–5 chondrocytes were used for each experiment. Primary chondrocytes were plated in 6-well plates and cultured in DMEM contained 10% FBS and 2 mM glutamine until 60% confluence. The doses of IL-1β, siRNA and K-80003 and the optimal time frame for each experiment were determined by preliminary studies. After transfection of RXRα SiRNA (Santa Cruz, sc-108077, 0.5 μM), or ERα SiRNA (Santa Cruz, sc-45949, 0.5 μM), or control siRNAs (Santa Cruz, sc-37007, 0.5 μM) with Lipofectamine 3000 (Invitrogen, L3000015) for 24 h, chondrocytes were incubated with vehicle (0.1% DMSO) or K-80003 (5 μM, 10 μM), or 17β-estradiol (10 nM) for 30 min, and then stimulated with IL-1β (10 ng/mL) for another 48 h before collected for RT-PCR, western bolt and immunofluorescent staining.

HEK293T cells were cultured in DMEM supplemented with 10% FBS, 100 U/mL penicillin G and 0.1 g/mL streptomycin in humidified 5% CO_2_ atmosphere at 37 °C. Cells were transfected pcDNA3.1-ERα and pcDNA3.1-RXRα using HiPerfect transfection reagent (Qiagen, 301704) and screened with G418 (0.3 mg/mL). An empty pCDNA3.1 mammalian expression vector was also transfected into control cells. Protein extracts were then separated from cells using RIPA lysis buffer.

### Competition Binding Assay

The binding affinity of K-80003 to ERα was detected by LanthaScreen TR-FRET ERα competitive binding kit (Invitrogen, A15887) following the manufacturer’s instructions. K-80003 (20 μM), fluorescent estrogen ligand (9 nM), or DMSO (negative control) was incubated with glutathione S-transferase (GST)-tagged ERα ligand binding domain (LBD) and terbium-labeled anti-GST antibody. The mixture was incubated at room temperature for 2 h. The fluorescent emission intensity at 520 nm was measured.

### Real-time quantitative PCR

Total RNA was extracted and analyzed using methods as previously described by our group^19,20^. The primer sequences for rat genes were as follows:

- ERα: 5′-ACTACCTGGAGAACGAGCCC-3′ (forward), 5′-CCTTGGCAGACTCCATGATC-3′ (reverse).
- MMP13: 5′-TGATGGCACTGCTGACATCAT-3′ (forward), 5′-TGTAGCCTTTGGAACTGCTT-3′ (reverse).
- ADAMTS-4: 5′-AGAGTCCGAACGAGTTTACG-3′ (forward), 5′-GTGCCAGTTCTGTGCGTC-3′ (reverse);
- TNFα: 5′-CATGATCCGAGATGTGGAACTGGC-3′ (forward), 5′-CTGGCTCAGCCACTCCAGC-3′ (reverse);
- IL-6: 5′-TGCCTTCTTGGGACTGATGTTG-3′ (forward), 5′-TGGTCTGTTGTGGGTGGTATCC-3′ (reverse);
- GAPDH: 5′-TGCCACTCAGAAGACTGTGG-3′ (forward), 5′-GTCCTCAGTGTAGCCCAGGA-3′ (reverse).

### Western blot

Protein isolation from primary chondrocytes was performed as we described previously^15^. Western blot was performed using a standard protocol^15,21^. Antibodies against the following proteins were used: rabbit anti-rat RXRα (Abcam, ab125001, dilution 1:1000); rabbit anti-rat ERα (Abcam, ab32063, dilution 1:1200); rabbit anti-rat p-p65 (Abcam, ab28856, dilution 1:2000); rabbit anti-rat p-IκBα (Abcam, ab32518, dilution 1:2,000); rabbit anti-rat HIF-2α (Abcam, ab199, dilution 1:2000); rabbit anti-rat GADPH (R&D, 2275-PC-100, dilution 1:3000).

### Co-immunoprecipitation (Co-IP) assay

Co-IP was done followed those described previously^22^.

### Data and statistical analysis

All immunohistochemical score were carried out blindly and independently by at least two investigators. Results are expressed as mean ± SEM. Data were analyzed using GraphPad Prism version 5.01. Data were analyzed by one-way analysis of variance (ANOVA) with Bonferroni’s and Dunnett’s post hoc multiple comparison tests. P < 0.05 was considered statistically significant.

## Results

### Cartilage protection by K-80003

**Figure 1:**
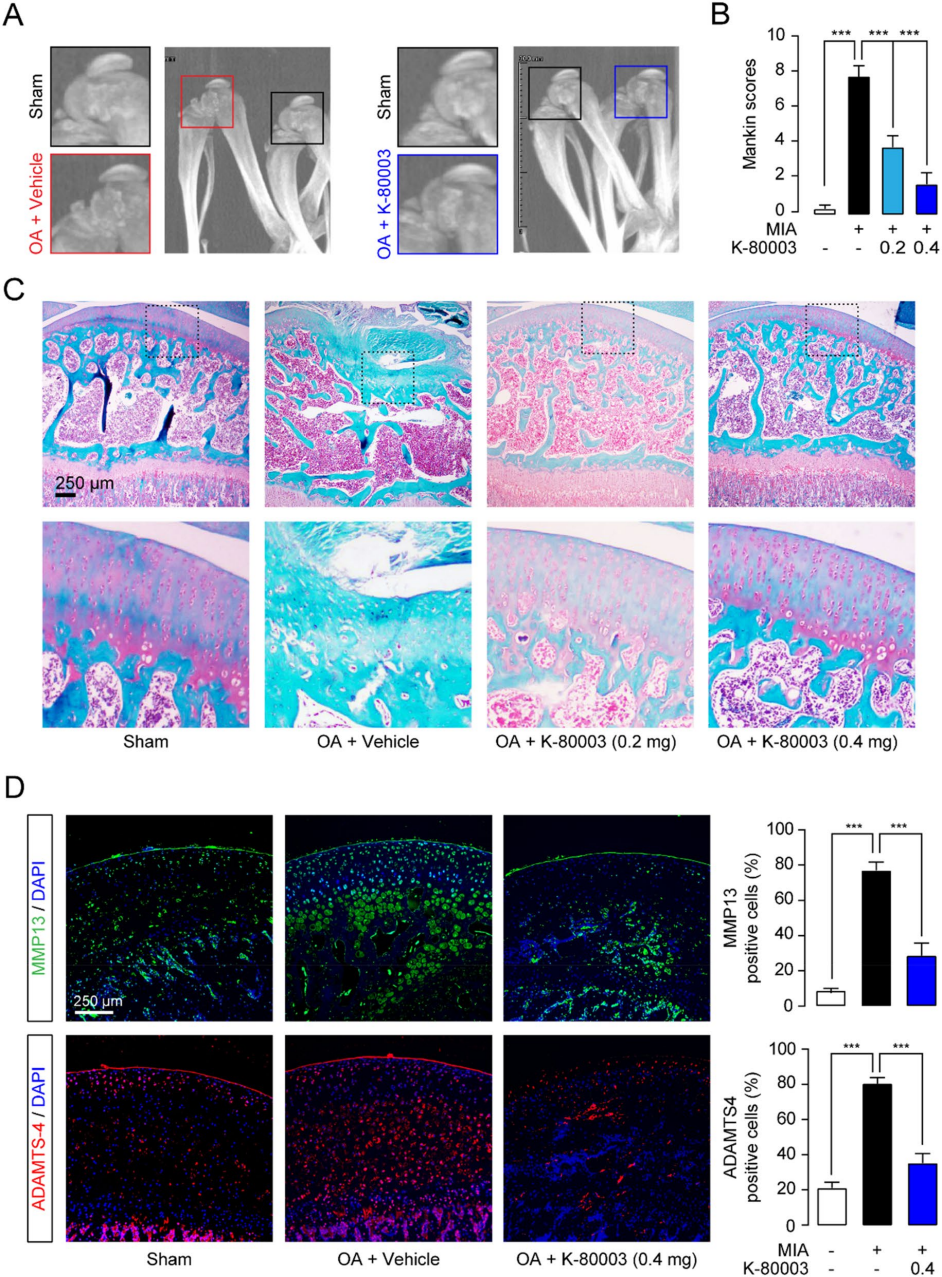
K-80003 prevented cartilage degeneration in OA rats. SD rats were injected intra-articularly (i.a*.*) with saline or MIA (1 mg, 20 μL) through the infrapatellar ligament of the hind knees, and were i.a. injected with vehicle or K-80003 (1 or 2 mg/kg, 20 μL) at days 7, 10, 14, 17, 21, 24 after MIA treatment. All animals were sacrificed 28 days after MIA injection, and knee joints were dissected for histopathological studies. (**A**) Representative radiographs of the knee joints of animals. (**B**, **C**) Safranin O and fast green staining of the tibia medial compartment of animals. Histopathological features were measured using the modified score of Mankin. (**D**) Immunofluorescence analyses and quantification of MMP-13 and ADAMTS-4 in the tibia medial compartment of animals. ***P < 0.001; n = 8.

Cartilage damage is the predominant consequence of OA, therefore, we first investigated whether K-80003 could maintained cartilage homeostasis in OA rats. OA was induced by intra-articular (i.a.) injection of MIA in the knee joint of rats. As indicated by radiographic analysis, vehicle-treated OA rats showed severe bone erosion in the femoral condyle and tibial plateau (Fig. 1A). K-80003 treatment significantly reduced the development of bone erosion (Fig. 1A). MIA induction low the expression of proteoglycan (Fig. 1B) and increased the number of MMP13 and ADAMTS-4 positive cells in cartilage in rats (Fig. 1C,D). However, K-80003 attenuated proteoglycan loss and the expressions of MMP13 and ADAMTS-4 (Fig. 1B,D). Taken together, these results indicate that K-80003 can protect articular cartilage during OA progression by downregulating cartilage catabolic enzymes.

### Attenuation of synovial inflammation by K-80003

**Figure 2:**
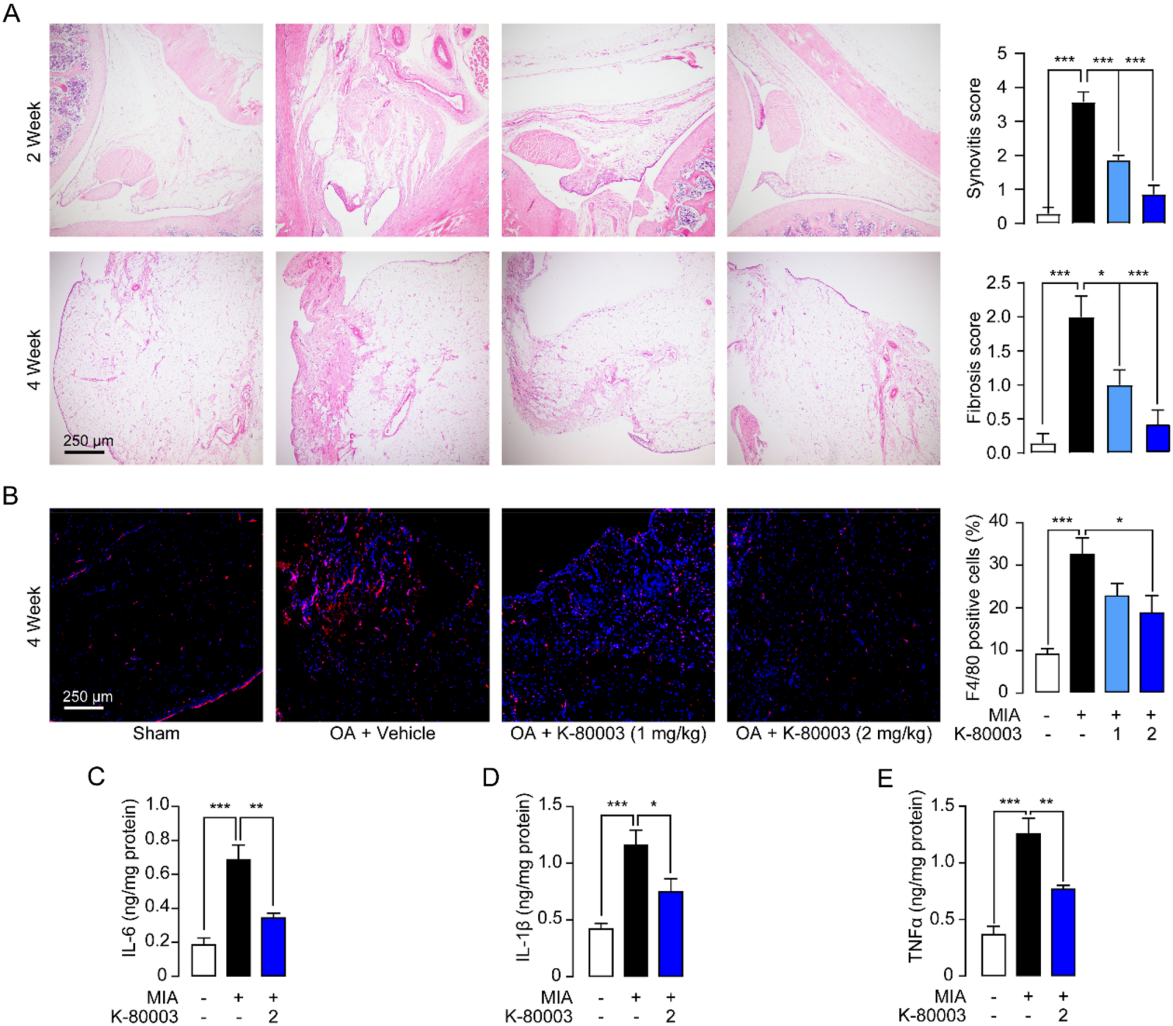
K-80003 regulates synovitis in OA rats. SD rats were injected intra-articularly (i.a.) with saline or MIA (1 mg, 20 μL) through the infrapatellar ligament of the hind knees, and were i.a*.* injected with vehicle or K-80003 (1 or 2 mg/kg, 20 μL) at days 7, 10, 14, 17, 21, 24 after MIA treatment. All animals were sacrificed 28 days after MIA injection, and synovium were dissected for ELISA and histopathological studies. (**A**) H&E staining of synovial membranes of animals at week 2 and 4. Synovium were measured by the synovitis score. (**B**) Immunofluorescence analyses and quantification of F4/80 in the tibia medial compartment of animals. (**C**–**E**) The concentrations of IL-6, IL-1β and TNF-α in homogenates of synovial membranes were measured by ELISA. *P < 0.05; **P < 0.01; ***P < 0.001; n = 8.

Next, we studied the effects of K-80003 on synovial inflammation, an important symptom of OA. Histological analysis showed that MIA elicited marked polymorphonuclear neutrophils (PMNs) infiltration and edema in the synovial tissues in week 2. As OA progresses, the infiltrate converted to fibroblasts and F4/80-positive monocytes/macrophage in week 4 (Fig. 2A,B). K-80003 reduced synovial edema at early phase and promoted the clearance of fibroblasts and monocytes/macrophage at late phase (Fig. 2A,B) in OA rats. MIA injection elicited a drastic increase in the expressions of IL-1β, IL-6, and TNFα in the synovial membranes, while K-80003 significantly suppressed the increment of these cytokines (Fig. 2C,E).

### Effects of K-80003 on osteoarthritic pain

**Figure 3:**
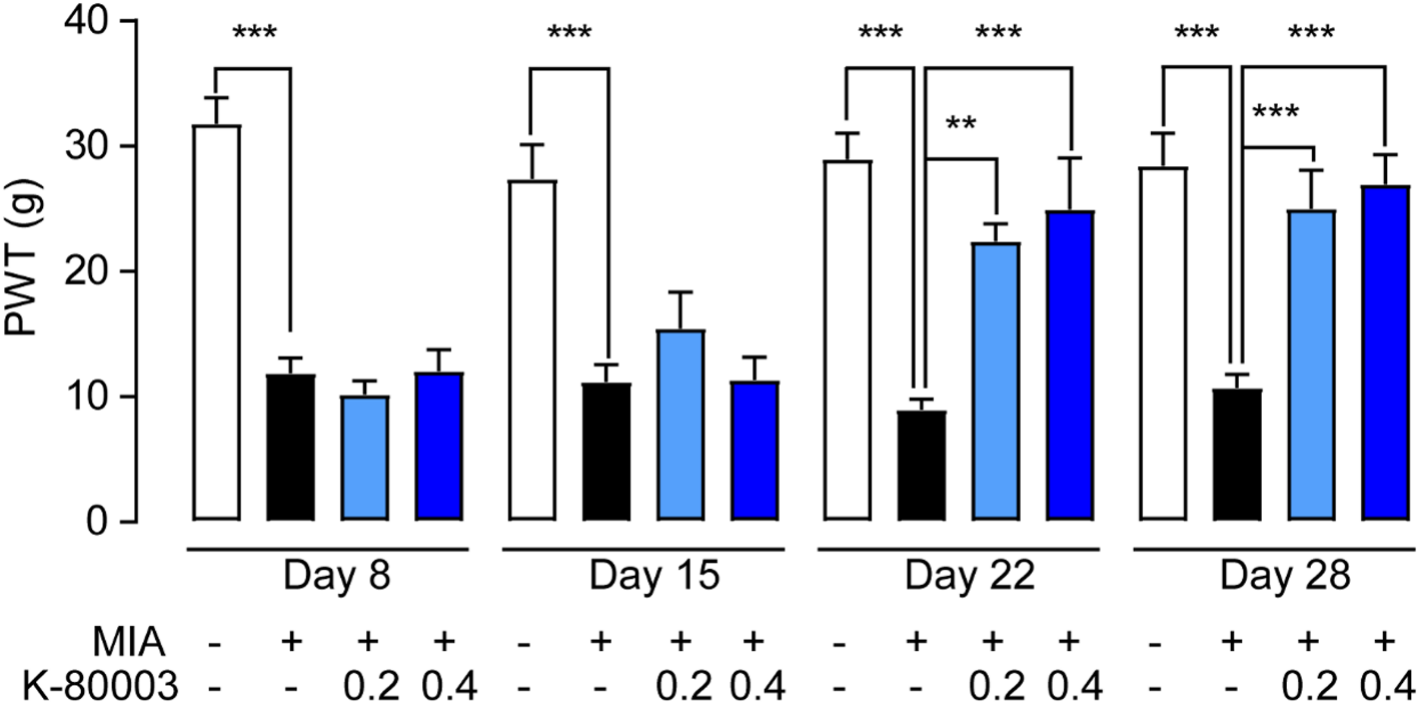
The analgesic effects of K-80003 on allodynia in OA rats. SD rats were injected intra-articularly (i.a.) with saline or MIA (1 mg, 20 μL) through the infrapatellar ligament of the hind knees, and were i.a. injected with vehicle or K-80003 (1 or 2 mg/kg) at days 7, 10, 14, 17, 21, 24 after MIA treatment. Tactile allodynia was tested on the hind paw in OA and sham rats at day 8, 15, 22 and 28, and measured the paw withdrawal threshold (PWT) values. *P < 0.05; **P < 0.01; ***P < 0.001; n = 8.

Pain is the most common reason that leads OA patients to seek medical intervention. We then tested the analgesic effects of K-80003 by measuring the secondary tactile allodynia in OA rats. On day 7, 14, 21 and 28 after OA surgery, the paw withdrawal threshold (PWT) (Fig. 3) was markedly decreased, indicating distal allodynia in OA rats. K-80003 had no significant analgesic effects in the early stages of OA (Day 8 and 15), but it increased the PWT at later time points (Day 22 and 28), suggesting its anti-allodynic property (Fig. 3).

### K-80003 enhances ERα signaling by dissociation of RXRα–ERα interaction

Encouraged by the in vivo anti-OA effects of K-80003, we further studied the signal pathways underlying the cartilage protective effects of K-80003 in vitro. RXRα normally regulates cellular processes through interacting other NRs that protective effects in OA, such as ERα, PPARα, PPARγ, LXRα and LXRβ^9,23–25^. Thus, we tested the influences of K-80003 on these NRs in primarily cultured rat chondrocytes. In chondrocytes challenged with the inflammatory stimulus, IL-1β. K-80003 significantly increased ERα expression, but it had poor effects on levels of PPARα, PPARγ, LXRα and LXRβ (Fig. S1). Moreover, receptors’ competitive binding assay showed that K-80003 did not bind to the ligand-binding domain (LBD) of ERα even at a high dose of 20 μM (Data not shown), suggesting that the effects of K-80003 were not due to directly binding to ERα.

**Figure 4:**
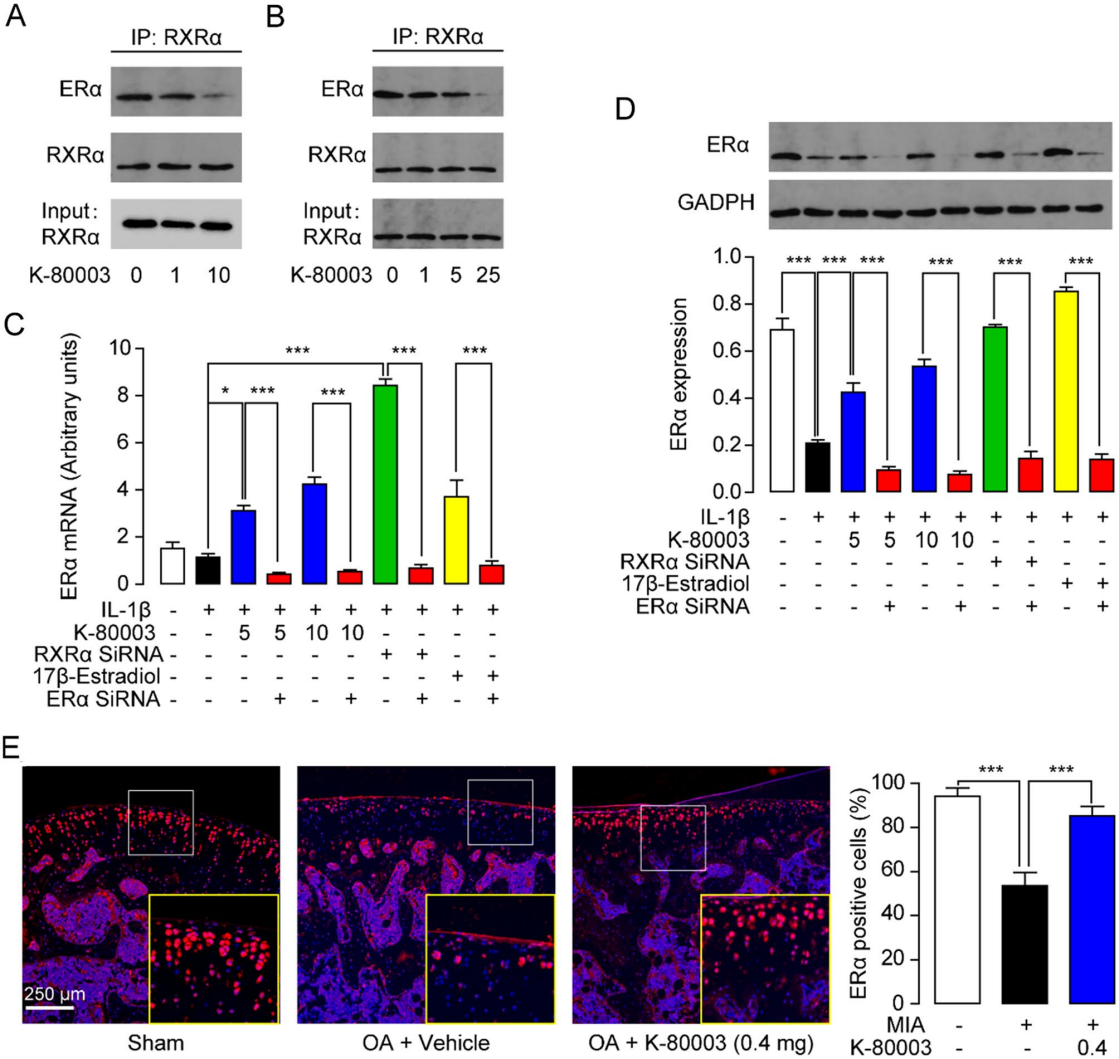
K-80003 enhances ERα signaling by dissociation of RXRα-ERα interaction. (**A**) HEK293T cells transfected with the RXRα and ERα plasmids were treated with K-80003 (1 μM, 10 μM) for 2 h and then analyzed by co-IP assays. (**B**) Primarily cultured rat chondrocytes were treated with K-80003 (1 μM, 5 μM, 25 μM) and analyzed by co-IP assays using anti-RXRα or anti-ERα antibody. Rat primary chondrocytes were transfected with RXRα SiRNA (0.5 μM), or ERα SiRNA (0.5 μM), or control siRNA (0.5 μM) with Lipofectamine 3000 for 24 h, following by incubation with vehicle, or K-80003 (5 μM, 10 μM) or 17β-estradiol (10 nM) for 30 min. Cells were then treated with IL-1β (10 ng/mL) for 48 h before analyzed expression of ERα by RT-PCR (**C**) and western-blot (**D**). (**E**) Immunofluorescence analyses and quantification of ERα in the tibia medial compartment of animals. ***P < 0.001; n = 5.

RXRα inhibits the activity of the ERα promoter through interacting with ERα^26^. Thus, we hypothesized that K-80003 may enhance ERα expression via modulating RXRα-ERα interaction. To test this hypothesis, we transfected RXRα and ERα expressing plasmids into HEK293 cells and studied the RXRα and ERα interaction using co-immunoprecipitation (Co-IP) assay. As shown in Fig. 4A, RXRα and ERα were co-precipitated from HEK293 cell extracts in all groups, suggesting that they do associate with each other. K-80003 dose-dependently decreased the level of RXRα-bound ERα, indicating that K-80003 dissociated RXRα from ERα. Additionally, similar effects of K-80003 on RXRα-ERα interaction were observed in primarily cultured rat chondrocytes (Fig. 4B).

To explore the influence of dissociation of RXRα-ERα complex on ERα signaling, we determined the expression of ERα by real-time PCR and western blot. Treatment with K-80003 or knockdown of endogenous RXRα by siRNA, greatly increased mRNA expressions of ERα in chondrocytes (Fig. 4C). Similarly, stimulation of chondrocytes with IL-1β significantly decreased protein expression of ERα, and K-80003 or RXRα siRNA restored disturbed expression of ERα in cells (Fig. 4D). Genetic down-regulation of ERα by specific siRNA prevented the effects of K-80003 and RXRα siRNA (Fig. 4C,D). Furthermore, we also confirmed the effects of K-80003 on ERα in vivo. ERα levels were down-regulated in cartilage in OA rats, while K-80003 promoted the expression of ERα (Fig. 4E). These results suggested that dissociation of RXRα from ERα enhanced ERα signaling.

### K80003-mediated upregulation of ERα suppresses NF-κB signaling

**Figure 5:**
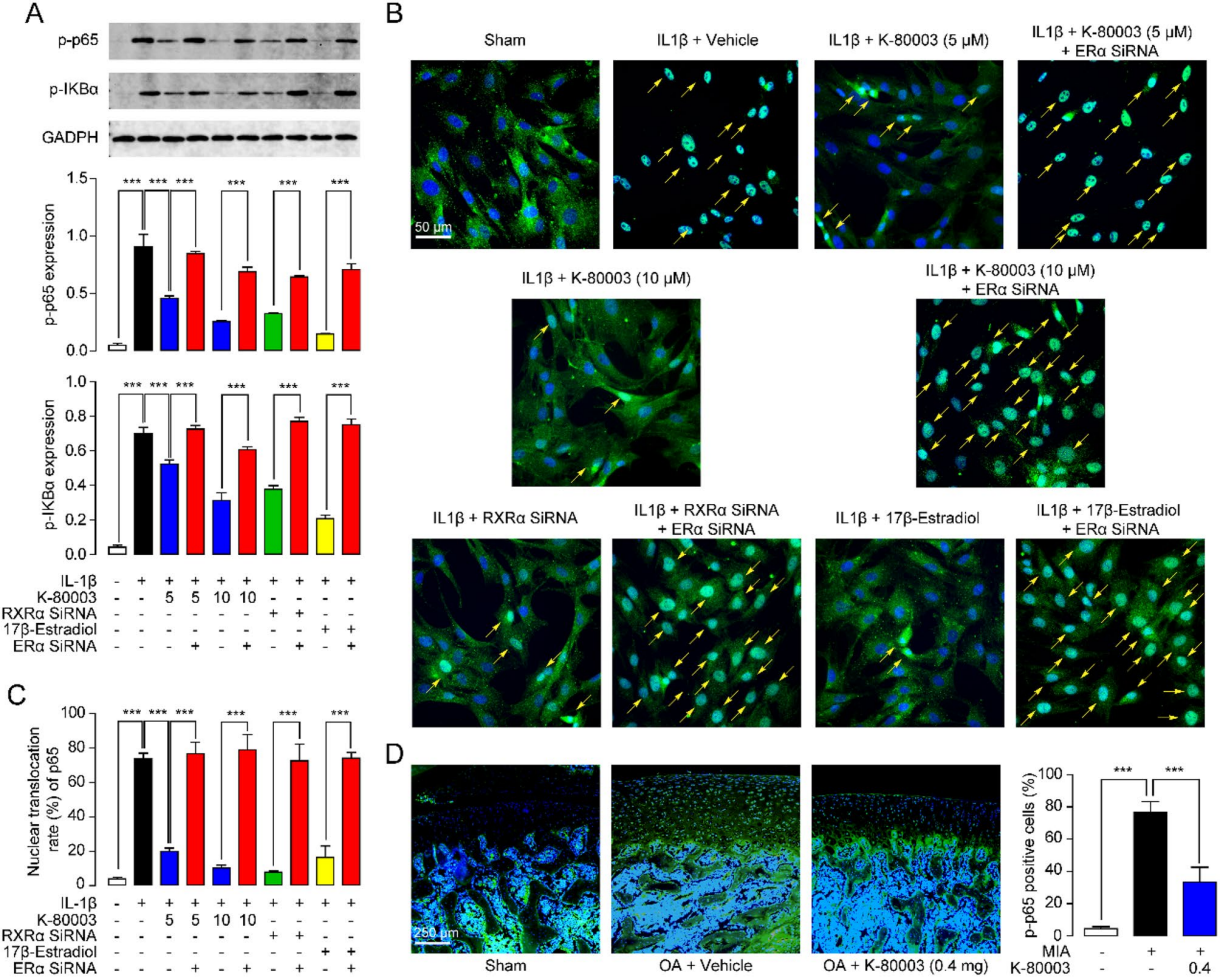
K-80003 reduced NF-κB activity in rat primary chondrocytes and joints. Rat primary chondrocytes were transfected with RXRα SiRNA (0.5 μM), or ERα SiRNA (0.5 μM), or control siRNA (0.5 μM) with Lipofectamine 3000 for 24 h, following by incubation with vehicle, or K-80003 (5 μM, 10 μM) or 17β-estradiol (10 nM) for 30 min. Cells were then treated with IL-1β (10 ng/mL) for 48 h. (**A**) Representative western-blot bands and quantification of of p-p65 and p-IκBα abundances in chondrocytes. (**B**) Confocal fluorescence imaging of p65 in chondrocytes. Green, p65; blue, nuclei; yellow arrows indicate nuclear translocation of p65. (**C**) Quantification of p65 nuclear translocation. Cells displaying p65 in nuclear localization are counted and expressed as a percentage of the total number of chondrocytes. (**D**) Immunofluorescence analyses and quantification of p-p65 in the tibia medial compartment of animals. ***P < 0.001; n = 8.

ERα regulates several signaling pathways involved in OA development, including NF-κB pathway. Growing evidence suggests that NF-κB pathway is closely associated with enhanced production of inflammatory cytokines or degrading enzymes^3^. We further explored the influence of K80003-mediated upregulation of ERα on NF-κB pathway in primarily cultured rat chondrocytes. As shown in Fig. 5A, IL-1β stimulation increased the phosphorylation of p65 (p-p65) and IκBα (p-IκBα). Treatment with K-80003 or ERα agonist 17β-estradiol, as well as knockdown of RXRα with siRNA reduced the protein levels of HIF-2α, p-p65 and p-IκBα (Fig. 5A). However, K-80003, 17β-estradiol and RXRα siRNA showed no effect in expressions of HIF-2α, p-p65 and p-IκBα in ERα siRNA-treated chondrocytes (Fig. 5A). Furthermore, we examined the effects of K-80003 on the nuclear translocation of p65. IL-1β stimulation enhanced the nuclear translocation of p65 in chondrocytes, while K-80003, 17β-estradiol and RXRα siRNA significantly suppressed such increment (Fig. 5B,C). ERα siRNA prevented the effects of K-80003 and RXRα siRNA on the p65 nuclear translocation in chondrocytes (Fig. 5B,C). Consistent with the data obtained from chondrocytes, p-p65 levels were up-regulated cartilage in OA rats, and K-80003 decreased the expressions of p-p65 (Fig. 5D).

**Figure 6:**
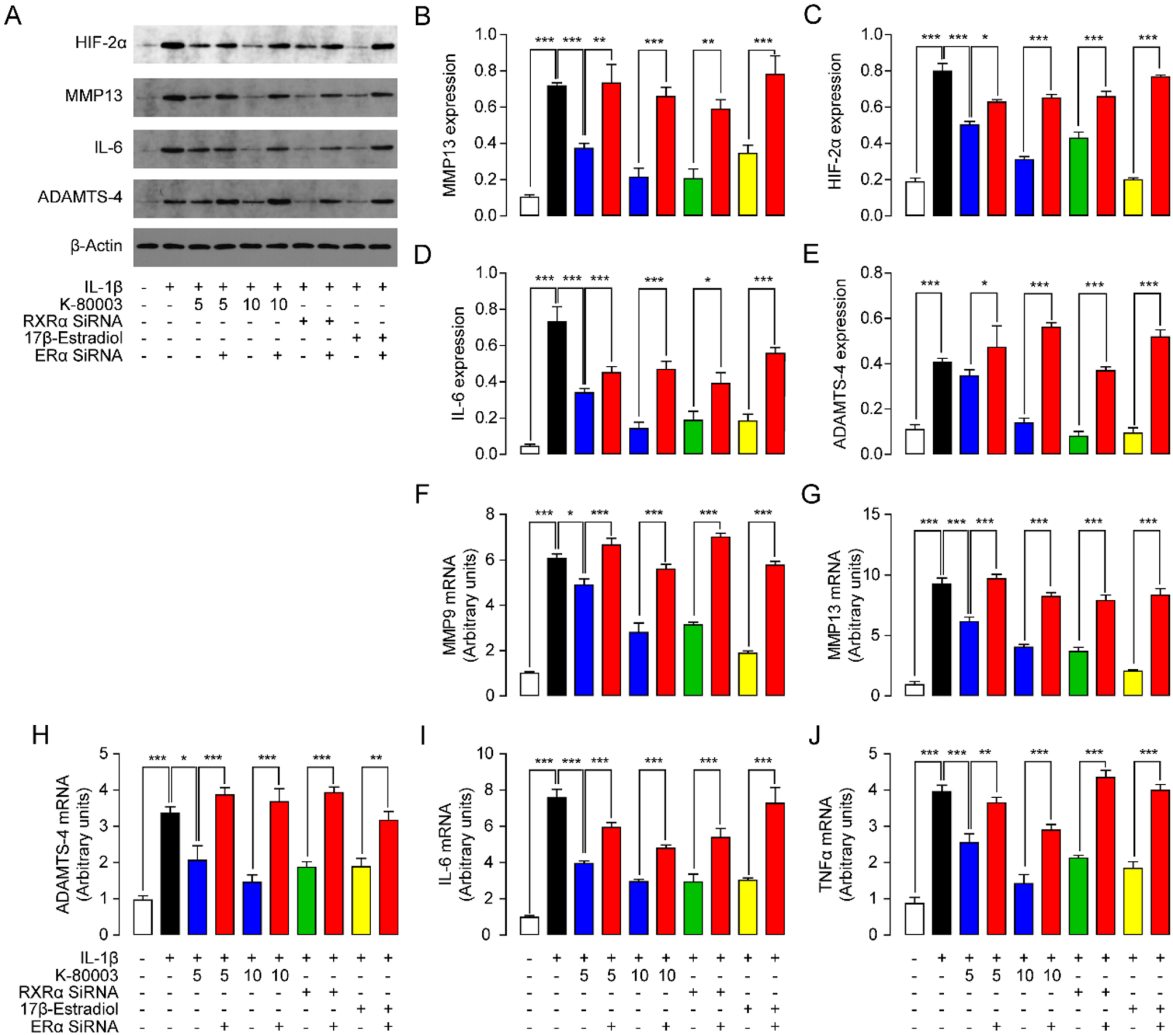
Effects of K-80003 on rat primary chondrocytes. Rat primary chondrocytes were transfected with RXRα SiRNA (0.5 μM), or ERα SiRNA (0.5 μM), or control siRNA (0.5 μM) with Lipofectamine 3000 for 24 h, following by incubation with vehicle, or K-80003 (5 μM, 10 μM) or 17β-estradiol (10 nM) for 30 min. Cells were then treated with IL-1β (10 ng/mL) for 48 h. (**A**–**D**) Representative western-blot bands and quantification of HIF-2α, MMP-13, ADAMTS-4 and IL-6 abundances in chondrocytes. (**E**–**J**) Effects of K-80003 on mRNA expression of MMP-9, MMP-13, ADAMTS-4, IL-6 and TNFα in chondrocytes. *P < 0.05; **P < 0.01; ***P < 0.001; n = 5.

Finally, we examined the expressions of serval proteinases and pro-inflammatory factors, including MMP9, MMP13, ADAMTS-4, IL-6 and TNFα, and hypoxia-inducible factor 2-alpha (HIF-2α), a NF-κB-related molecule that is essential for OA development^3^. As expected, incubation of IL-1β with chondrocytes increased the levels of MMP9, MMP13, ADAMTS-4, IL-6 and TNFα (Fig. 6). Dissociation of RXRα from ERα induced by K-80003 or RXRα siRNA, as well as activation of ERα by 17β-estradiol, suppressed such increment (Fig. 6). K-80003, 17β-estradiol and RXRα siRNA had no effects in expressions of MMP9, MMP13, ADAMTS-4, IL-6 and TNFα in ERα deficient cells (Fig. 6).

## Discussion

Osteoarthritis (OA), a progressive and degenerative joint disorder, has attracted increasing attention in recent years. There are no consistently effective therapeutic approaches to prevent cartilage degradation or slow its progression. Here, we reported that RXRα modulator K‐80003 prevented inflammatory and destructive responses in a rat model of osteoarthritis. Mechanistically, K‐80003 protected cartilage through dissociating RXRα from ERα, and subsequently inhibiting NF‐κB signaling via ERα pathway.

The most important finding presented here is that RXRα modulators may represent a new strategy to treat OA. Previous studies showed that RXRα may involve in the pathogenesis of OA. RXRα agonist SR11237 decreased expression of aggrecan and increased expression of MMP13 in chondrocytes^27^. However, treatment of rats with SR11237 resulted in shorter long bones, irregular ossification and dysmorphic growth plate^28^. In this study, we demonstrated that RXRα modulator K‐80003 significantly decreased MMP13 and ADAMTS-4 expressions, thus prevented cartilage degeneration and reduced the development of bone erosion in OA (Fig. 1). In addition, K-80003 significantly reduced recruitment of macrophages, the main source of pro-inflammatory factors during OA. Subsequently, it attenuated synovial inflammation and suppressed production of pro-inflammatory cytokines (Fig. 2). Furthermore, K-80003 prolonged PWT at later time points (Day 21–28), but not at early stage of OA (Day 7–14), indicating that K-80003 may alleviate osteoarthritic pain via alleviating chronic inflammation and cartilage damage, rather than directly inhibiting the noxious inputs to the brain.

There is increasing evidence that ERs signaling is associated with OA development. ERs gene mutation and down-regulation are linked with OA severity^8,29^. Reduced ER signaling has been postulated to contribute to the prevalent of OA among postmenopausal women. Activation of ERs in joint tissues by estrogen or selective estrogen receptor modulators (Bazedoxifene, Raloxifene and Levormeloxifene) inhibited degeneration of articular cartilage and improved synovial inflammation and joint pain^9^. Previous studies showed that RXRα inhibited the activity of the ERα promoter through interacting with ERα^26^. In agreement with previous studies, our results showed that ERα associated with RXRα and RXRα modulator K-80003 interrupt this interaction (Fig. 4). Although how K-80003 modulates RXRα-ERα interaction remains to be explored, we hypothesized that K-80003 might bind to RXRα ligand‐binding domain (RXRα-LBD), subsequently changed the interface of RXRα-LBD and affected the affinity towards Erα. on^30^. Additionally, RXRα-ERα complex dissociation induced by K-80003 or knockdown of endogenous RXRα attenuated RXRα’s suppression on ERα expression (Fig. 4) and consequently inhibited the downstream NF‐κB pathway (Fig. 5). Furthermore, ERα SiRNA blocked the effects of K-80003 and RXRα SiRNA on NF‐κB pathway, by, indicating that the impact of K-80003 and RXRα SiRNA on NF‐κB pathway is at least partially through interacting with ERα (Fig. 5).

NF-κB pathway, activated in chondrocytes in OA, plays an important role in OA pathophysiology, including inflammation, chondrocytes survival, proliferation, and differentiation^3^. Growing evidence suggests that suppression of NF-κB activity prevents the degeneration of articular cartilage during OA development^3,31^. ERα may regulate NF‐κB signaling via several distinct pathways. ERα can inhibit NF‐κB signaling through interacting with estrogen response element (ERE) or other transcription factors in the presence of ligands. Previous studies showed that activation of ERα with 17β-estradiol suppressed NF-κB activity and subsequently, inhibited IL-1β-induced nitric oxide production^32^. On the other hand, ERα can be activated by TGF-β/SMAD and Wnt/β-catenin signaling pathways in the absence of ligands. SMADs 3/4elicit estrogen responses by interacting with ER^10^. In this study, we found that K80003-mediated upregulation of ERα inhibited NF-κB activity in a ligand-independent manner (Figs. 4, 5). However, how ERα interacts with NF‐κB in the absence of the ligand remains unclear. We hypothesized that TGF-β/SMAD and Wnt/β-catenin signaling might involve in the K80003-ERα-mediated NF-κB inhibition, further studies are needed to elucidate the underlying mechanisms by which ERα impacts NF‐κB pathway. Moreover, K-80003 may also suppresse NF‐κB signaling through the non-genomic pathways. Both ERα and RXRα can translocate from the nucleus to the cytoplasm in response to inflammation to modulate important biological processes^30,33,34^. However, whether and how ERα and RXRα act in the cytoplasm in OA are still unknown. A more thorough investigation of the cytoplasmic effects of K-80003 and the possible interaction between ERα and RXRα in the cytoplasm is needed to answer these questions.

In summary, our results demonstrated that K‐80003 increased ERα expression by binding to RXRα and dissociating RXRα from ERα. Enhanced ERα further suppressed the NF‐κB signaling and protected cartilage in OA. When combined, current studies suggested that targeting RXRα‐ERα interaction by RXRα modulators may lead to a novel therapeutic approach for OA.

## Supplementary Information

Supplementary Information.

Supplementary Figures.

## Data Availability

Research data supporting the results of this paper will be provided by corresponding author at reasonable request.
