# Supplementary material for: The retinoid X receptor α modulator K-80003 suppresses inflammatory and catabolic responses in a rat model of osteoarthritis
Source: Sci Rep. 2021 Aug 20;11:16956. doi: 10.1038/s41598-021-96517-y (PMC8379249; doi:10.1038/s41598-021-96517-y)
Supplement: Supplementary file 1 — Supplementary Information. [file 41598_2021_96517_MOESM1_ESM.pdf]

NOTE: Please save this file locally before filling in the table, DO NOT work on the file within your internet browser as changes will not be saved. Adobe Acrobat Reader (available free here) is recommended for completion.

ARRIVE

The ARRIVE guidelines 2.0: author checklist

The ARRIVE Essential 10

These items are the basic minimum to include in a manuscript. Without this information, readers and reviewers cannot assess the reliability of the findings.

Item		Recommendation	Section/line number, or reason for not reporting
**Study design**	1	For each experiment, provide brief details of study design including:	
		a. The groups being compared, including control groups. If no control group has been used, the rationale should be stated.	Section 2.2
		b. The experimental unit (e.g. a single animal, litter, or cage of animals).	Section 2.2
**Sample size**	2	a. Specify the exact number of experimental units allocated to each group, and the total number in each experiment. Also indicate the total number of animals used.	Section 2.2
		b. Explain how the sample size was decided. Provide details of any *a priori* sample size calculation, if done.	line 138-139
**Inclusion and exclusion criteria**	3	a. Describe any criteria used for including and excluding animals (or experimental units) during the experiment, and data points during the analysis. Specify if these criteria were established *a priori*. If no criteria were set, state this explicitly.	line 135-138
		b. For each experimental group, report any animals, experimental units or data points not included in the analysis and explain why. If there were no exclusions, state so.	line 135-138
		c. For each analysis, report the exact value of *n* in each experimental group.	line 135-138
**Randomisation**	4	a. State whether randomisation was used to allocate experimental units to control and treatment groups. If done, provide the method used to generate the randomisation sequence.	Section 2.12
		b. Describe the strategy used to minimise potential confounders such as the order of treatments and measurements, or animal/cage location. If confounders were not controlled, state this explicitly.	Section 2.2
**Blinding**	5	Describe who was aware of the group allocation at the different stages of the experiment (during the allocation, the conduct of the experiment, the outcome assessment, and the data analysis).	Section 2.12
**Outcome measures**	6	a. Clearly define all outcome measures assessed (e.g. cell death, molecular markers, or behavioural changes).	Section 2.6
		b. For hypothesis-testing studies, specify the primary outcome measure, i.e. the outcome measure that was used to determine the sample size.	Section 3.1-3.3
**Statistical methods**	7	a. Provide details of the statistical methods used for each analysis, including software used.	Section 2.12
		b. Describe any methods used to assess whether the data met the assumptions of the statistical approach, and what was done if the assumptions were not met.	Section 2.12
**Experimental animals**	8	a. Provide species-appropriate details of the animals used, including species, strain and substrain, sex, age or developmental stage, and, if relevant, weight.	Section 2.2
		b. Provide further relevant information on the provenance of animals, health/immune status, genetic modification status, genotype, and any previous procedures.	Section 2.2
**Experimental procedures**	9	For each experimental group, including controls, describe the procedures in enough detail to allow others to replicate them, including:	Section 2.2
		a. What was done, how it was done and what was used.	Section 2.2
		b. When and how often.	Section 2.2
		c. Where (including detail of any acclimatisation periods).	
		d. Why (provide rationale for procedures).	Section 2.2
**Results**	10	For each experiment conducted, including independent replications, report:	Section 2.12
		a. Summary/descriptive statistics for each experimental group, with a measure of variability where applicable (e.g. mean and SD, or median and range).	
		b. If applicable, the effect size with a confidence interval.	Figure legends
