# Supplementary material for: The retinoid X receptor α modulator K-80003 suppresses inflammatory and catabolic responses in a rat model of osteoarthritis
Source: Sci Rep. 2021 Aug 20;11:16956. doi: 10.1038/s41598-021-96517-y (PMC8379249; doi:10.1038/s41598-021-96517-y)
Supplement: Supplementary file 2 — Supplementary Figures. [file 41598_2021_96517_MOESM2_ESM.docx]

The retinoid X receptor α modulator K-80003 suppresses inflammatory and catabolic responses in a rat model of osteoarthritis

Hua Li, Xiaofan Li, Boyu Yang, Junnan Su, Shaofang Cai, Jinmei Huang, Tianfu Hu, Lijuan Chen, Yaping Xu, Yuhang Li *

*, Corresponding author: [yuhangli@fjirsm.ac.cn](mailto:yuhangli@fjirsm.ac.cn) (YL)


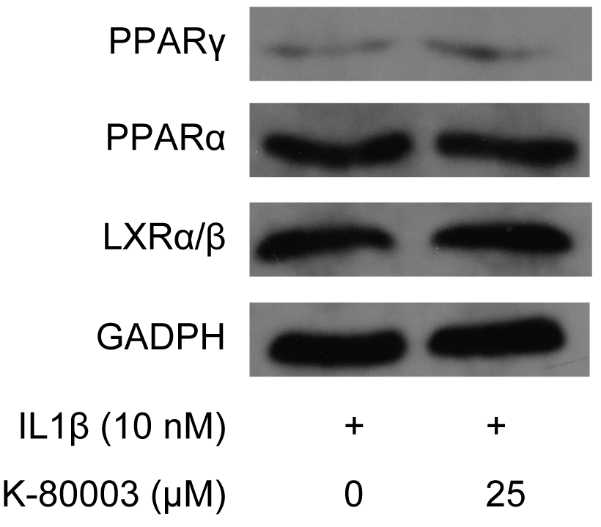


**Figure S1. Effects of K-80003 on NRs in rat primary chondrocytes.** Rat primary chondrocytes were incubated with vehicle or K-80003 for 30 min, then treated with IL-1β (10 ng/mL) for 48 h. Representative western-blot bands of NRs abundances in chondrocytes.


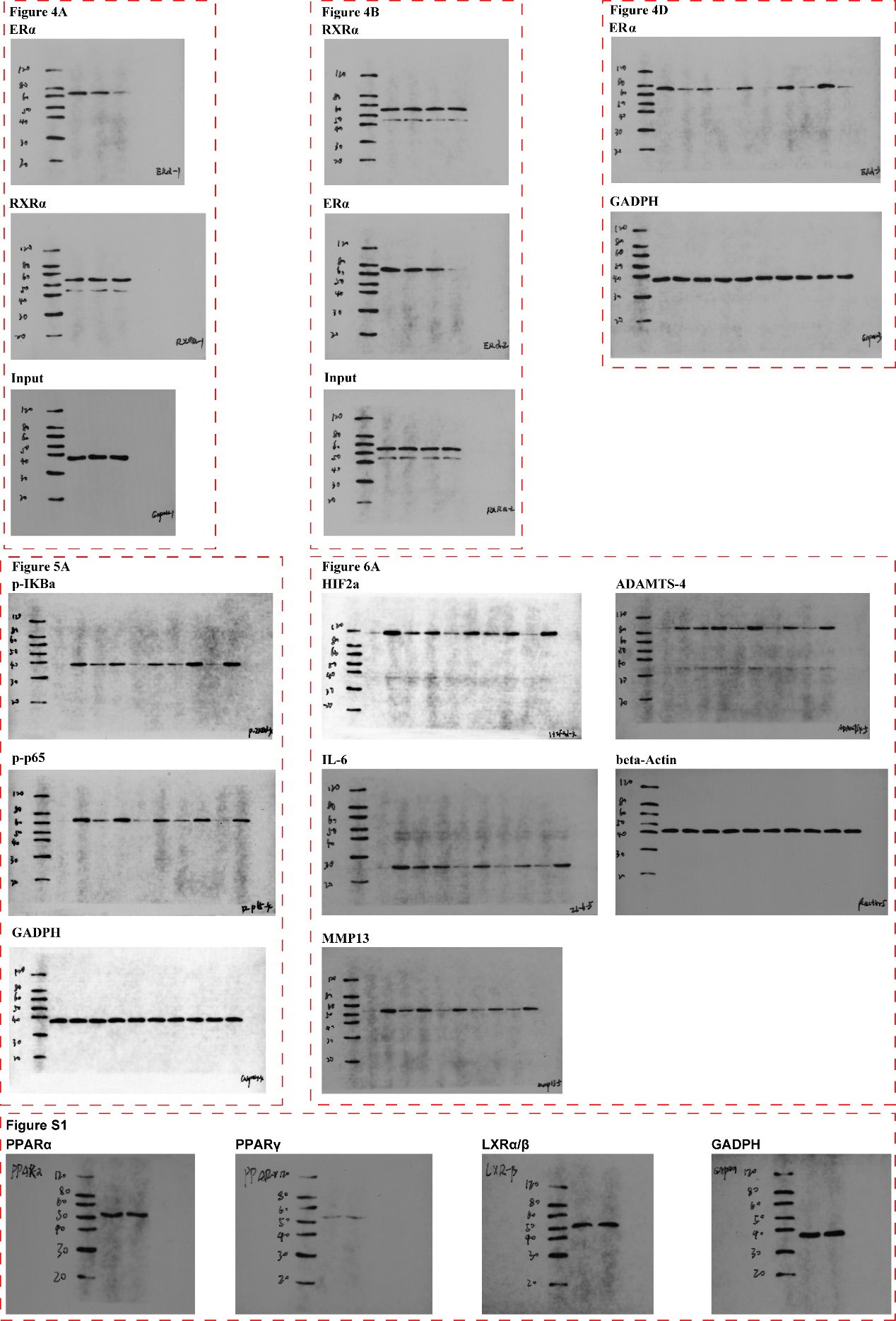


**Supplementary figure S2.** Images of full-length blots presented in the figures.
